# Polyphonia: detecting inter-sample contamination in viral genomic sequencing data

**DOI:** 10.1093/bioinformatics/btae698

**Published:** 2024-12-02

**Authors:** Lydia A Krasilnikova, Christopher H Tomkins-Tinch, Alton C Gayton, Stephen F Schaffner, Sabrina T Dobbins, Adrianne Gladden-Young, Katherine J Siddle, Daniel J Park, Pardis C Sabeti

**Affiliations:** Howard Hughes Medical Institute, Chevy Chase, MD 20815, United States; Infectious Disease and Microbiome Program, Broad Institute, Cambridge, MA 02142, United States; Department of Organismic and Evolutionary Biology, Harvard University, Cambridge, MA 02138, United States; Infectious Disease and Microbiome Program, Broad Institute, Cambridge, MA 02142, United States; Department of Virology, Harvard Medical School, Harvard University, Cambridge, MA 02138, United States; Infectious Disease and Microbiome Program, Broad Institute, Cambridge, MA 02142, United States; Department of Organismic and Evolutionary Biology, Harvard University, Cambridge, MA 02138, United States; Department of Immunology and Infectious Diseases, Harvard T.H. Chan School of Public Health, Harvard University, Boston, MA 02115, United States; Infectious Disease and Microbiome Program, Broad Institute, Cambridge, MA 02142, United States; Department of Molecular Biology and Microbiology, Tufts University Graduate School of Biomedical Sciences, Boston, MA 02111, United States; Department of Molecular Microbiology and Immunology, Brown University, Providence, RI 02912, United States; Infectious Disease and Microbiome Program, Broad Institute, Cambridge, MA 02142, United States; Howard Hughes Medical Institute, Chevy Chase, MD 20815, United States; Infectious Disease and Microbiome Program, Broad Institute, Cambridge, MA 02142, United States; Department of Organismic and Evolutionary Biology, Harvard University, Cambridge, MA 02138, United States; Department of Immunology and Infectious Diseases, Harvard T.H. Chan School of Public Health, Harvard University, Boston, MA 02115, United States; Massachusetts Consortium for Pathogen Readiness, Boston, MA 02115, United States

## Abstract

**Summary:**

In viral genomic research and surveillance, inter-sample contamination can affect variant detection, analysis of within-host evolution, outbreak reconstruction, and detection of superinfections and recombination events. While sample barcoding methods exist to track inter-sample contamination, they are not always used and can only detect contamination in the experimental pipeline from the point they are added. The underlying genomic information in a sample, however, carries information about inter-sample contamination occurring at any stage. Here, we present Polyphonia, a tool for detecting inter-sample contamination directly from deep sequencing data without the need for additional controls, using intrahost variant frequencies. We apply Polyphonia to 1102 SARS-CoV-2 samples sequenced at the Broad Institute and already tracked using molecular barcoding for comparison.

**Availability and implementation:**

Polyphonia is available as a standalone Docker image and is also included as part of viral-ngs, available in Dockstore. Full documentation, source code, and instructions for use are available at https://github.com/broadinstitute/polyphonia.

## 1 Introduction

Inter-sample contamination can occur through many means, including processing errors or sample aerosolization. Amplicon sequencing is especially vulnerable due to the high number of amplicon copies produced in each PCR cycle. Ramifications of inter-sample contamination can be significant: it can alter minor alleles more commonly or consensus-level alleles at the extreme, potentially affecting analysis of viral variants, superinfections, recombination events, within-host evolution, and transmission and outbreak reconstruction.

Inter-sample contamination can be tracked experimentally by barcoding samples with spike-ins such as synthetic DNA spike-ins (SDSIs) (Lagerborg *et al.* 2022) or External RNA Control Consortium (ERCC) RNA standard spike-ins (Jiang *et al.* 2011, Matranga *et al.* 2014). SDSIs are short (∼200 bases) DNA spike-ins added during amplicon sequencing. ERCCs are longer (hundreds to thousands of bases) RNA spike-ins added during metagenomic sequencing and fragmented alongside sample RNA. Both are designed to be used qualitatively.

In practice, spike-ins cannot be used to detect contamination that occurred prior to their addition; they also cannot be used in retrospect if not added. Viral genomic information, on the other hand, tracks samples from collection through sequencing without alterations to the sequencing pipeline or additional controls. Existing tools to detect contamination in genomic data include Squeegee (Liu *et al.* 2022), decontam (Davis *et al.* 2018), and DeconSeq (Schmieder and Edwards 2011); these tools do not, however, identify same-species inter-sample contamination. Existing tools to specifically infer same-species inter-sample contamination from genomic data alone include, for human genomic data, ART-DeCo (Fiévet *et al.* 2019) and ContEst within GATK (Cibulskis *et al.* 2011), which detect unexpected allelic ratios and are not applicable to viruses; and, for viral metagenomic data, Cont-ID (Rollin *et al.* 2023), which requires an external control which must be included in sequencing; no broadly applicable, computational-only tool for viruses exists.

Here, we present Polyphonia, a tool for detecting inter-sample contamination between samples of the same target organism using deep sequencing data (SARS-CoV-2 deep sequencing data from a plate of samples from patients with COVID-19, e.g.). We compare Polyphonia to spike-ins in 1102 COVID-19 samples generated at the Broad Institute in a high-throughput sequencing setting with rigorous quality-control practices. These samples were already tracked with SDSIs or ERCC spike-ins, allowing a comparison measure of inter-sample contamination detection.

## 2 Implementation

**Figure 1.**
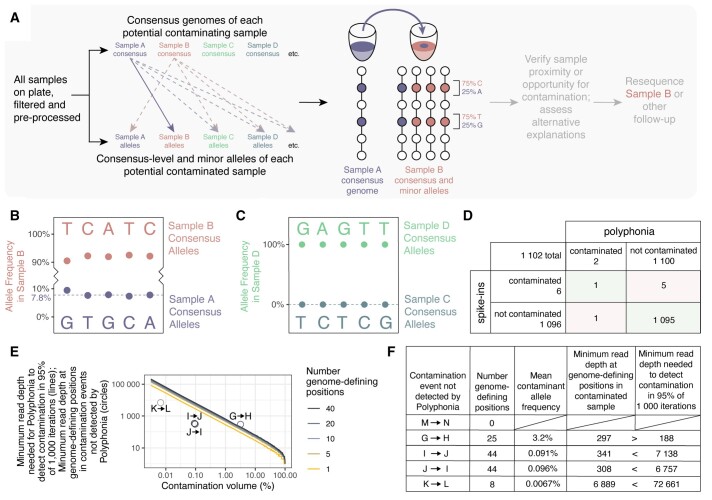
Inter-sample contamination detection by Polyphonia. (A) All samples are compared pairwise to identify putative contaminating and contaminated samples. Sample A is flagged as contaminating Sample B if Sample A’s consensus alleles appear as minor alleles in Sample B at genome-defining positions where their consensus genomes differ. (B) Contamination by Sample A was detected in Sample B. Alleles and allele frequencies at genome-defining positions are shown. Sample B consensus-level alleles are indicated above; Sample A consensus-level alleles, identical to Sample B minor alleles, are indicated below. Median contaminating allele frequency is indicated by a dashed line. (C) No putative contamination by Sample C was detected in Sample D. Figure is as in B. (D) Results of validating Polyphonia against spike-ins in 1102 COVID-19 samples. (E) Minimum read depth required to detect contamination at all genome-defining positions, thereby enabling detection by Polyphonia with default parameters, in 95% of 1000 iterations. The four instances of contamination not detected by Polyphonia with ≥1 genome-defining positions are shown by circles. (F) Minimum read depths needed to detect contamination and observed minimum read depths at genome-defining positions in the five instances of contamination not detected by Polyphonia.

Pairs of samples are compared to detect potential inter-sample contamination in which a contaminating sample’s consensus genome appears in the minor alleles of a contaminated sample (Fig. 1A). For example, Sample A is marked as putatively contaminating neighboring Sample B because Sample A’s consensus alleles appear as minor alleles in Sample B at genome-defining positions where the consensus genomes of Sample A and Sample B differ (Fig. 1B). In contrast, Sample D shows no evidence of contamination by neighboring Sample C: Sample D does not contain the consensus alleles of Sample C as minor alleles at genome-defining positions (Fig. 1C).

If plate maps are provided, each sample is compared to samples in neighboring wells, its row, its column, or the full plate; otherwise, all samples are compared. By default, only positions with read depth ≥100 and samples with 95% of the genome covered at read depth ≥100 are included. By default, contamination must be detected in all genome-defining positions, of which there must be at least three. These cut-offs produced the highest concordance with spike-ins in this dataset.

The outputs are plate visualizations and a table describing putative inter-sample contamination.

## 3 Methods

### 3.1 Sample collection, sequencing, and computational processing

Eighty-five 96-well plate batches of nasopharyngeal or nasal samples from patients positive for COVID-19 were collected between April 2020 and June 2021, then barcoded with ERCC spike-ins (Jiang *et al.* 2011, Matranga *et al.* 2014) and sequenced using metagenomic sequencing as described in Lemieux *et al.* (2021), and Tomkins-Tinch *et al.* (2021) (24 batches, 1600 samples), or barcoded with SDSIs (Lagerborg *et al.* 2022) and sequenced using amplicon sequencing as described in Petros *et al.* (2022), Siddle *et al.* (2022), and Turbett *et al.* (2023) (61 batches, 5116 samples) (Supplementary data). Samples were processed at the Broad Institute and include but are not limited to those described in Lemieux *et al.* (2021), Tomkins-Tinch *et al.* (2021), Petros *et al.* (2022), Siddle *et al.* (2022), and Turbett *et al.* (2023). Each batch contained 24–92 clinical samples (median 91, mean 79). All sequenced samples were processed in viral-ngs (Park 2015, https://viral-pipelines.readthedocs.io) on Terra as described in Lemieux *et al.* (2021), Tomkins-Tinch *et al.* (2021), Petros *et al.* (2022), Siddle *et al.* (2022), and Turbett *et al.* (2023). Allele frequencies were calculated using LoFreq call v2.1.5 (Wilm *et al.* 2012) in the isnvs_lofreq workflow in viral-ngs (Park 2015) by dividing allele read counts (DP4) by read depths (DP).

### 3.2 Sample filtering

Six thousand seven hundred and sixteen samples were filtered to those with ≥95% of the genome (≥26 913 bases of NC_045512.2) covered to read depth ≥100 (3350 samples) and with neither of the top two spike-ins appearing as one of the top two spike-ins in ≥3% of samples and the expected spike-in appearing with ≥1000 reads as one of the two highest read count spike-ins (2306 samples). After filtering, 1102 samples remained.

### 3.3 Identification of contamination events

Samples were processed with Polyphonia in viral-ngs on Terra with masking of positions with potential sequencing errors (265 positions) as described in De Maio (2020) (https://virological.org/t/masking-strategies-for-sars-cov-2-alignments/480) and otherwise default settings.

Putative contamination events called by spike-ins were defined as instances where ≥2% of spike-in reads were attributed to the spike-in with the highest read count other than the expected spike-in.

Only putative contamination events involving neighboring wells (above, below, left, right, or diagonal) were examined.

The following samples are described: Sample A: USA/MA-MASPHL-00091/2020; B: USA/MA-MASPHL-00098/2020; C: USA/MA-MGH-00437/2020; D: USA/MA-MGH-00444/2020; E: USA/MA-Broad_CRSP-01315/2021; F: USA/MA-Broad_CRSP-01323/2021; G: USA/RI-CDCBI-RIDOH_01534/2021; H: USA/RI-CDCBI-RIDOH_01535/2021; I: USA/CT-CDCBI-CRSP_01382/2021; J: USA/VT-CDCBI-CRSP_01374/2021; K: USA/MA-MASPHL-00025/2020; L: USA/MA-MASPHL-00024/2020; M: USA/MA-MASPHL-00117/2020; N: USA/MA-MASPHL-00116/2020.

## 4 Results: comparing Polyphonia and spike-ins in SARS-CoV-2 clinical samples

Both Polyphonia and spike-ins were used to trace inter-sample contamination in 1102 COVID-19 clinical samples. Samples were either processed with amplicon sequencing and spiked with SDSIs (Lagerborg *et al.* 2022) or processed with metagenomic sequencing and spiked with ERCC spike-ins (Jiang *et al.* 2011, Matranga *et al.* 2014). Six putative inter-sample contamination events involving neighboring wells were detected by spike-ins and two were detected by Polyphonia, with one detected by both (Fig. 1D).

One putative inter-sample contamination event was detected by both ERCC spike-ins and Polyphonia (Fig. 1B): the consensus genome of contaminant Sample A matched five minor alleles in contaminated Sample B with otherwise identical consensus genomes. Polyphonia estimated 7.8% contamination volume (median contaminating allele frequency at genome-defining positions, range 7.4%–9.4%). Contamination was detected by ERCC spike-ins with an estimated 17.8% contamination volume (proportion spike-in reads in Sample B attributed to the spike-in added to Sample A).

Polyphonia detected one putative inter-sample contamination event not identified by SDSI spike-ins. Contamination by Sample E was detected in Sample F. Sample F contained 21 base-substitution minor alleles and one deletion, all appearing at consensus level in Sample E; their consensus genomes were otherwise identical. Median contaminating allele frequency in Sample F was 4.7% (3.2%–12.0%). Superinfection was unlikely, as neither genome had other exact matches (100% identity and 100% coverage or no mutations) in a MegaBLAST search against the NCBI Betacoronavirus database (accessed 31 January 2024) or in a SARS-CoV-2 UShER (Turakhia *et al.* 2021) tree (accessed 1 February 2024). Resequencing Sample F from the point of collection could distinguish contamination and superinfection.

Five putative inter-sample contamination events were detected by spike-ins but not by Polyphonia. In one, ERCC spike-in values suggest that Sample M contaminated Sample N with estimated 24.6% contamination volume. However, their two genomes were identical, making detection of contamination by Polyphonia impossible. In the other four contamination events not detected by Polyphonia, some genome-defining positions had heterozygosity consistent with contamination while other genome-defining positions had no heterozygosity: in these cases, it is possible that with higher read depth, Polyphonia would have identified these contamination events. In the first of these four contamination events, Sample H had heterozygosity compatible with contamination by Sample G in 17 of 25 genome-defining positions; the other eight positions had no heterozygosity. Including positions without heterozygosity (contaminating allele frequency 0%), median contaminating allele frequency was 1.7% (0%–16.5%), compared with contaminating SDSI spike-in frequency 7.7%. In a similar case, SDSI spike-in values suggest that samples I and J contaminated each other, with estimated contamination volume 46.0% in one direction (J to I) and 48.0% in the other (I to J). Of 44 genome-defining positions, eight had heterozygosity in Sample I consistent with contamination by Sample J, with median contaminating allele frequency 0% (0%–1.0%), while 16 had heterozygosity in Sample J consistent with contamination by Sample I, with median contaminating allele frequency 0% (0%–0.6%); the remaining genome-defining positions had no heterozygosity. Finally, Sample K was detected to have contaminated Sample L with 16.0% contaminating ERCC spike-in frequency. Of eight genome-defining positions, two showed evidence of contamination by K in L.

To examine the four contamination events between nonidentical genomes detected by spike-ins but not by Polyphonia, we identified the minimum read depth needed for Polyphonia to detect contamination (for all genome-defining positions to contain at least one contaminating read) in 95% of 1000 simulations (Fig. 1E). In three of the four contamination events, the minimum read depth at genome-defining positions was less than that needed (Fig. 1E and F). In the fourth, the detected contaminating allele read counts at genome-defining positions were 4, 0, 0, 0, 2, 0, 0, and 0 and the mean frequency of contaminating alleles was 0.0067%—low enough for stochastic effects and low enough to have minimal practical impact on downstream analyses.

## 5 Discussion

We describe Polyphonia, a method to detect inter-sample contamination using viral deep sequencing data. A sample is marked as putatively contaminated by another sample if the contaminating sample’s consensus genome appears in the minor alleles of the contaminated sample at positions where the two consensus genomes differ.

Across 1102 COVID-19 samples, Polyphonia identified two putative inter-sample contamination events, one confirmed by spike-ins and one likely occurring before spike-in addition, demonstrating the utility of Polyphonia in the common instance when quality measures are not available and even as a supplement to them. Using spike-ins, we were able to trace five additional contamination events not identified by Polyphonia, including one in which the genomes were identical, precluding detection by Polyphonia, and four with low-frequency contamination that we hypothesize would be detected by Polyphonia with higher read depth.

In general, Polyphonia will not detect inter-sample contamination if it is at consensus level; if the contaminant and contaminating genomes are identical or, with default settings, have fewer than three differences; if either sample has less than the minimum required genome coverage; or if genome-defining positions are not covered in sufficient read depth. Polyphonia also requires that all samples contain the same target organism: SARS-CoV-2, e.g. in a plate of samples from patients with COVID-19. Polyphonia is not suitable, e.g. for detecting inter-sample contamination between complex wastewater sequences or samples from patients with symptoms of unknown etiology. Spike-ins fill all these gaps in functionality: unlike Polyphonia, spike-ins allow detection of inter-sample contamination independent of the identity, quality, or diversity of the sequenced genomes. On the other hand, assuming that allele frequencies are constant throughout the sample preparation and sequencing process, Polyphonia follows a sample across its full lifetime, allowing detection of inter-sample contamination when spike-ins are absent. To take full advantage of their respective strengths, we recommend a two-pronged approach, with spike-ins and Polyphonia complementing each other.

Polyphonia joins a universe of contamination detection tools. If one were interested in detecting contamination from reagents or lab environments, one might use Squeegee (Liu *et al.* 2022), decontam (Davis *et al.* 2018), or DeconSeq (Schmieder and Edwards 2011), while if one were interested in detecting inter-sample contamination in human genomic data, one might use ART-DeCo (Fiévet *et al.* 2019) or ContEst within GATK (Cibulskis *et al.* 2011). If one needed to detect inter-sample contamination in viral genomic sequencing data and had not yet processed the samples, and had the resources to do additional processing, we recommend the addition of SDSIs (Lagerborg *et al.* 2022) for amplicon sequencing or ERCC spike-ins (Jiang *et al.* 2011, Matranga *et al.* 2014) for metagenomic sequencing, as well the alien external control used for Cont-ID (Rollin *et al.* 2023). If the sequencing batch is of the same target organism (a plate of samples from COVID-19 patients, e.g.), we recommend then applying Polyphonia to the resultant viral genome sequencing data, whether or not other tools were used, especially as Polyphonia can capture inter-sample contamination from before the addition of spike-ins. Indeed, in situations where spike-ins and the Cont-ID alien external control were not added or inter-sample contamination occurred before sample processing, Polyphonia is the only available tool.

## Supplementary Material

btae698_Supplementary_Data

## Data Availability

The sequence data underlying this article are available in the Sequence Read Archive (SRA) with BioSample accessions provided in Supplementary data. Additionally, plate maps and spike-in data are available in Supplementary data.
